# Customized Silicone Ocular Prosthesis for Post-exenteration Rehabilitation: A Case Report

**DOI:** 10.7759/cureus.81283

**Published:** 2025-03-27

**Authors:** Meghna D Agarwal, Girija Dodamani, Ashwini Pungle, Ashwini Salunke, Vini Mistry, Arun Dodamani, Seema Gupta

**Affiliations:** 1 Department of Prosthodontics, Jawahar Medical Foundation’s Annasaheb Chudaman Patil Memorial Dental College, Dhule, IND; 2 Department of Public Health Dentistry, Jawahar Medical Foundation’s Annasaheb Chudaman Patil Memorial Dental College, Dhule, IND; 3 Department of Orthodontics, Kothiwal Dental College and Research Centre, Moradabad, IND

**Keywords:** customized, exenteration, orbital, prosthesis, silicone

## Abstract

Ocular loss due to malignancies, trauma, or congenital anomalies can lead to significant aesthetic and psychological challenges. Orbital exenteration, a radical surgical procedure involving the removal of the eye and surrounding structures, leaves patients with severe facial deformities, impairing their self-esteem and social interactions. Prosthetic rehabilitation plays a vital role in restoring facial symmetry and improving the quality of life for such individuals. This case report presents the prosthetic rehabilitation of a 57-year-old male patient who underwent orbital exenteration due to squamous cell carcinoma. Despite undergoing surgery in 2009, the patient was not referred for a prosthetic solution at the time, leading to years of social withdrawal and psychological distress. Upon referral to the Department of Prosthodontics, various treatment options were discussed, and a custom silicone ocular prosthesis was chosen based on the patient’s preference for an economical and non-invasive solution. The fabrication process involved a systematic approach, starting with primary and secondary impressions to accurately capture the anatomic contours of the defect. A wax pattern was created for trial fitting and aesthetic evaluation, ensuring a close match with the patient’s natural eye. The final prosthesis was processed using room-temperature vulcanized silicone, which provided superior aesthetics, flexibility, and comfort. The prosthesis was designed to be retained using natural anatomical undercuts, eliminating the need for adhesives or external support. The patient exhibited significant psychological and social improvement following prosthesis insertion. The custom ocular prosthesis successfully restored facial aesthetics, improved self-confidence, and facilitated social reintegration. This case underscores the importance of early prosthetic referral and highlights the impact of a multidisciplinary approach in post-exenteration rehabilitation, ensuring optimal functional and aesthetic outcomes for patients with orbital defects.

## Introduction

The eyes are often described as the “mirror of the soul,” playing a fundamental role in human interaction, facial aesthetics, and emotional expression. Beyond their function as organs of vision, they contribute significantly to an individual’s identity and self-esteem. Unfortunately, various pathological conditions and traumatic incidents can lead to eye loss, resulting in profound physical and psychological challenges [1]. The causes of ocular loss may include carcinoma, severe ocular trauma, painful blind eyes, congenital defects, or conditions such as sympathetic ophthalmia [2]. In such cases, surgical interventions such as evisceration, enucleation, or orbital exenteration are necessary to manage these conditions and prevent further complications [3].

Eye loss can cause facial asymmetry, impair social interactions, and negatively impact an individual’s confidence and mental well-being. Restoring both form and function is a priority for postoperative rehabilitation. One of the most effective approaches to rehabilitation is the fabrication of a custom-made ocular prosthesis that aims to mimic the natural eye as closely as possible, restore facial harmony, and improve the patient’s quality of life [4,5].

The fabrication of an ocular prosthesis involves several critical steps, including impression making, wax pattern development, color matching, and final prosthesis fabrication [5]. Achieving a lifelike appearance requires precision and expertise because the prosthesis must replicate the color, size, orientation, and natural movements of the remaining eye [6]. Prosthodontists play a crucial role in ensuring that facial prostheses are aesthetically pleasing and functionally acceptable. The challenge lies in developing a prosthesis that seamlessly integrates with the surrounding tissues, providing the patient with a sense of normalcy [7].

In more complex cases, such as orbital exenteration, where the eye and surrounding structures, including the eyelids, muscles, and portions of the bony orbit, are surgically removed, the rehabilitation process becomes even more intricate [8]. This procedure is typically performed to manage malignant tumors, aggressive infections, and severe traumas. However, it results in major cosmetic and functional deformities, making prosthetic rehabilitation essential. In such cases, an orbital prosthesis, which restores the missing tissues along with the eye, is fabricated using biocompatible materials, such as acrylic resins and silicone elastomers. Retentive aids such as adhesives, osseointegrated implants, and magnets are often employed to enhance prosthesis stability [9].

Successful rehabilitation following ocular loss or orbital exenteration requires a multidisciplinary approach that involves ophthalmic surgeons, maxillofacial prosthodontists, and psychologists. Ophthalmic surgeons play a crucial role in preserving anatomical structures that can aid in prosthetic retention, whereas prosthodontists ensure the fabrication of a well-fitting and cosmetically acceptable prosthesis. Early patient counselling and education regarding available prosthetic options can significantly reduce psychological distress and facilitate smoother rehabilitation [8,9].

This case report provides a guide for presenting a comprehensive step-by-step approach to the simplified fabrication of an ocular prosthesis, outlining the key procedures and materials used in the process. By following a structured methodology, prosthodontists can ensure optimal aesthetic and functional outcomes, thereby enhancing the overall quality of life of individuals who have undergone ocular surgery. Through advancements in material science and digital technologies, modern ocular prostheses continue to evolve, offering patients improved comfort, durability, and realism.

## Case presentation

**Figure 1:**
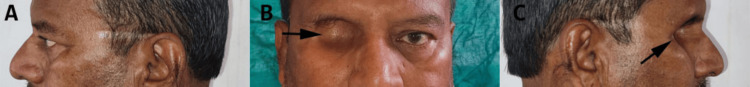
Initial examination of the exenterated socket.

A 57-year-old male patient was referred to the Department of Prosthodontics at the Jawahar Medical Foundation’s Annasaheb Chudaman Patil Memorial Dental College and Hospital, Dhule, Maharashtra, for cosmetic rehabilitation following exenteration of his right ocular socket due to squamous cell carcinoma. Exenteration was performed in 2009; however, the patient was not referred for cosmetic rehabilitation at the time and was unaware of the possibility of prosthetic intervention. Over the years, he experienced significant psychological and social distress due to facial defects, which led to social withdrawal and forced him to leave his job due to compromised vision (Figure 1).

Upon referral to the prosthodontics department, the patient expressed his desire for an economical and user-friendly prosthesis that would restore facial aesthetics while allowing ease of placement and removal through tactile sensation. Considering his requirements, the patient was given various treatment options, including an implant-retained prosthesis. However, the patient declined the latter option because of the cost and surgery involved. A silicon eye prosthesis for the missing eye was then planned for the patient. The patient was informed of the treatment, and consent was obtained for the proposed treatment plan. An ocular prosthesis was planned using silicone, which provides a more lifelike appearance and allows better integration with the surrounding skin.

Clinical procedure

Initial Examination and Preparation

Before initiating the impression-making process, the exenterated socket was thoroughly cleaned by irrigation with cold saline solution and drying with sterile cotton pellets. To ensure ease of impression removal, the patient’s eyebrows and eyelashes on the affected side were lubricated using petroleum jelly.

Primary Impression

**Figure 2:**
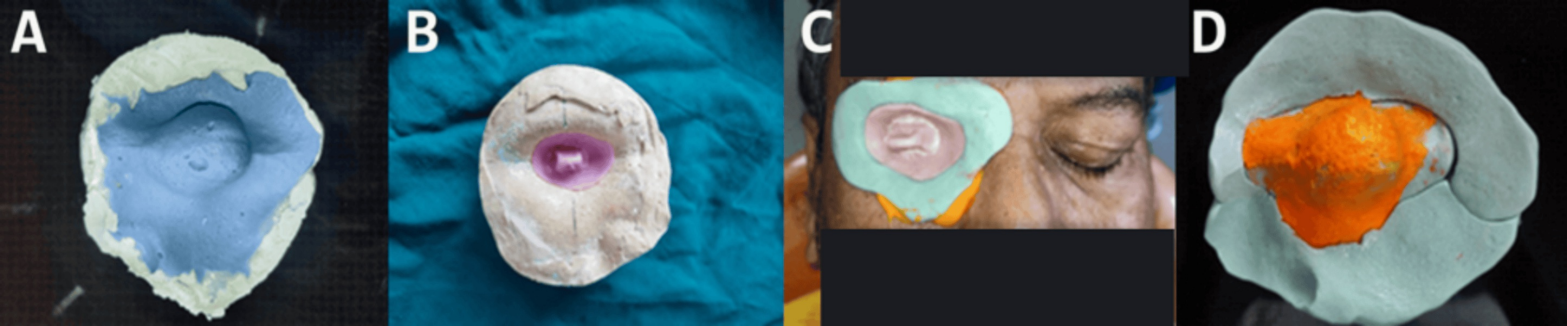
(A) Fabrication of final impression. (B) Primary impression. (C) Acrylic custom tray. (D) Final impression with putty and light body silicone material. (E) Final impression.

The primary impression was recorded using a slow-setting alginate impression material (Algitex, DPI, India) supported by an overlying plaster mold. Once set up, the impression was poured into a dental stone (Ultrastone, Kalabhai, India) to create a detailed facial model. Dental stone was chosen for its durability, strength, and ability to accurately replicate fine anatomical details of the defect. This primary cast served as the foundation for the subsequent steps in prosthesis fabrication (Figure 2, Panel A).

Secondary Impression

After obtaining the primary cast, a separating medium was applied to prevent adhesion during the impression-making process. A wax spacer was placed to ensure adequate space for the prosthesis, mimicking soft-tissue contours. A custom impression tray was fabricated using a cold-cure acrylic resin, which provided improved support and allowed for a more precise secondary impression (Figure 2, Panel B). For the secondary impression, light-body silicone was used to capture intricate details of the ocular socket, ensuring an accurate fit. A putty material was applied alongside the light-body silicone to provide structural support during the impression setting (Figure 2, Panel C). This secondary impression (final impression) helped create a highly detailed final cast (Figure 2, Panels D, E).

Final Cast Preparation and Prosthesis Fabrication

**Figure 3:**
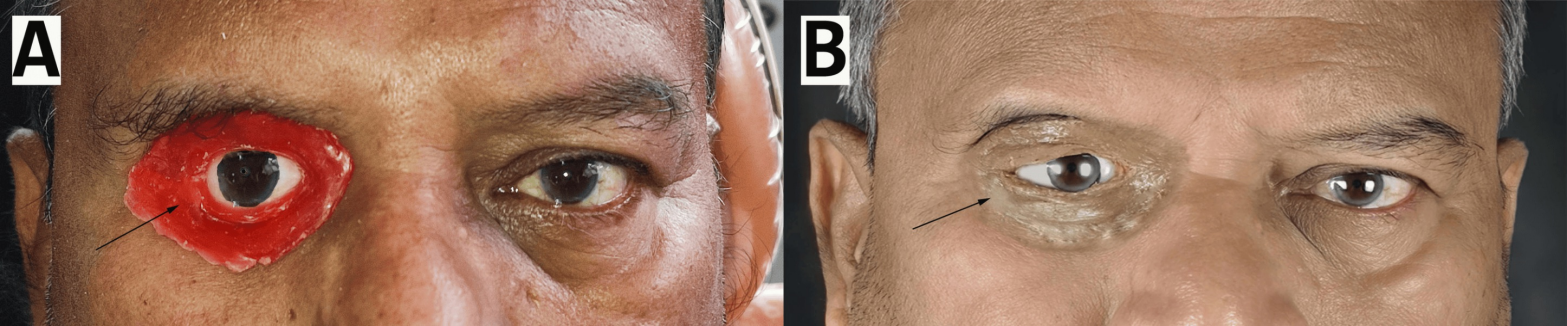
(A) Wax trial. (B) Final prosthesis.

The secondary impression was poured using high-strength dental stone to produce the final cast, which replicated the exact anatomical contour of the defect. The final cast served as the working model for the prosthesis fabrication. A wax pattern was then sculpted on the final cast to evaluate fit, projection, and alignment of the ocular prosthesis. Iris color was selected and customized to match the patient’s natural eye. During the wax trial phase, the prosthesis was positioned in the socket to assess fit and comfort (Figure 3, Panel A).

Adjustments were made to iris position, size, and shape to achieve optimal aesthetics and symmetry. The patient was involved in the evaluation to ensure satisfaction before final processing.

Processing the Ocular Prosthesis

Once the wax pattern was finalized, the prosthesis was processed using room-temperature vulcanized (RTV) silicone, which was selected for its flexibility, biocompatibility, and natural appearance. The processing involved flasking, where the wax pattern was encased in a flask and dental stone was poured around it to create a mold. This was followed by wax elimination, where the mold was heated to remove the wax, leaving behind a cavity corresponding to the prosthesis shape. This was followed by silicone processing, in which RTV silicone was mixed, pigmented to match the patient’s skin tone, and injected into the mold. Curing was done, where the mold was left to cure at room temperature, allowing the silicone to set and take its final shape. After curing, the prosthesis was retrieved, polished, and detailed. Additional surface characterization was performed to mimic natural scleral veins and subtle color variations to ensure a lifelike appearance.

Final Fitting and Delivery

The completed prosthesis was inserted into the patient’s ocular socket and evaluated for fit, retention, and aesthetics. The silicone material provided a seamless blend with the surrounding tissues, making the margins less visible (Figure 3, Panel B). The prosthesis was designed to gain retention from the underlying bony and soft tissue undercuts, eliminating the need for adhesives or spectacles for support. The lightweight nature of silicone ensured its comfort and ease of handling.

Patient Education and Follow-Up

The patient was educated about the correct placement, removal, and maintenance of the prosthesis. Although manual dexterity was required for positioning, the patient was able to learn the process through practice. Routine follow-up was scheduled to assess adaptation, comfort, and any necessary adjustments.

## Discussion

In cases of orbital exenteration, prosthetic rehabilitation plays a crucial role in restoring facial aesthetics and improving psychological well-being. The most commonly used materials for facial prosthesis fabrication are acrylic elastomers and silicone elastomers. Acrylic acid offers several advantages such as good longevity, excellent aging properties, low cost, ease of processing, and better adherence to spectacle frames. It generally requires minimal maintenance, and the rigidity of acrylic resin is rarely an issue because the tissue bed under the prosthesis is usually immovable [10]. Benson [11] proposed a technique for fabricating a custom-made acrylic resin ocular prosthesis. Sharma et al. [12] proposed a simplified approach to fabricate an ocular prosthesis using acrylic resin in a 40-year-old man.

Silicone materials, on the other hand, are increasingly preferred owing to their superior marginal adaptation and more lifelike appearance than acrylic. However, silicone prostheses tend to be more expensive and lack the ability to chemically or mechanically bond with the eyeglass frames, which can present challenges in terms of retention. Despite these challenges, the aesthetic benefits of silicone make it a popular choice for many patients [13].

The fabrication of silicone prosthesis has a significant positive impact on a patient’s quality of life as it provides functional and aesthetic benefits. Implant-retained prostheses, while offering superior retention and overall treatment satisfaction, often result in higher costs and require more invasive procedures. In contrast, adhesive-retained prosthesis offer a more cost-effective and non-invasive treatment alternative [14].

However, one of the major drawbacks of silicone prosthesis, particularly adhesive-retained prosthesis, is the need for frequent aftercare. Over time, these prostheses can experience discoloration and breakdown at their margins. These issues are often observed in adhesive-retained prosthesis and can impact both the retention and overall aesthetic appearance. As a result, patients may require the prosthesis to be refabricated, which can lead to additional time and expense in maintaining the prosthetic solution [14,15].

While durable, acrylic prostheses tend to be heavier and less adaptable to facial contours. In addition, hiding prosthesis margins using spectacles is often impractical. In contrast, silicone prostheses offer superior aesthetics owing to their natural texture, flexibility, and ability to merge seamlessly with the patient’s skin. Retention of an ocular prosthesis in our case could be achieved using bony and soft tissue undercuts within the defect site, eliminating the need for adhesives. The flexible nature of silicone allows it to conform closely to the contours of the socket, aiding retention. The prosthesis was molded precisely according to the patient’s anatomy, ensuring a snug fit without requiring adhesives. The patient learned to manually position and secure the prosthesis through tactile feedback and practice. This method is cost-effective and functionally satisfactory.

## Conclusions

Successful rehabilitation of our patient with a custom silicone ocular prosthesis significantly improved his self-esteem, social interactions, and overall quality of life. The lightweight and aesthetically pleasing nature of the prosthesis provides a practical and economical solution to meet patient needs. This case highlights the importance of timely prosthetic referral and intervention for post-exenteration patients to enhance their psychosocial well-being and reintegration into society. A collaborative approach between ophthalmologists and maxillofacial prosthodontists is essential for achieving optimal outcomes. By combining their expertise, these specialists can significantly improve the quality of life of patients with orbital defects, facilitating their quick reintegration into daily activities, and enhancing both their physical appearance and psychological well-being. Effective teamwork ensures that patients receive the best possible care, resulting in both functional and cosmetic restoration after orbital exenteration.
